# Remarks on Convulsions of Children, as They Occur during Paroxysms of Intermittent Fever

**Published:** 1849-09

**Authors:** P. A. Allaire

**Affiliations:** Aurora, Illinois


### ARTICLE II.

*Remarks on the Convulsions of Children, as they occur during Paroxysms of Intermittent Fever.* By P. A. ALLAIRE, M. D., of Aurora, Illinois.

The disordered conditions and diseases of the brain and nervous system, may well be considered as among the most interesting to the medical attendant, as they are in some of their forms the most appalling to the friends of the sufferer; while our knowledge of them is, perhaps, less precise, in consequence of the many and peculiar difficulities which attend their investigation. Of the functions of many organs and their physical condition, we may learn something by our senses, but of the brain and spinal cord next to nothing, and we can learn nothing of the patient in many cases. There is, also, another fact which increases the obscurity of this class of affections, viz., the dissimilarity of post mortem appearances in some cases where the symptoms are the same.

The convulsions of children, occurring during attacks of ague, I do not now remember to have seen specially noticed; but it is a frequent complication in our miasmal district, and where not correctly managed may be a fatal one. All the cases observed by me have been between the ages of six months and six years, but a large majority of them was under three years of age. I have not remarked that one six years of age was more frequently attacked than the other. A deviation from the healthy circulation of the blood in the brain, I have thought would rationally account for this disturbed state of the nervous functions, and that deviation would seem to be produced by pressure. The normal circulation in this organ in children is more active than in adults, which causing a greater impressibility, will account for the ease with which irritation may here be lighted up, and it is, in fact, the reason of so many serious brain fevers occuring among them, which are originally but sympathetic affections. But that there is in this form of convulsion no cause of a reflex character, seems certain from the many signs of direct congestion present, caused by the increased force of the heart's action, and the subsequent spasm of the platysma muscles, compressing the jugular veins.

The attack occurs usually, shortly after the accession of the hot stage, occasionally before it is fairly established, and with but few, often no premonitory symptoms. Sometimes an unusual restlessness may be observed during the latter part of the cold stage, but generally not until this subsides can we perceive any sign of its approach, then, if the child dozes, he may start, cry out or clinch his hands; or if awake and up, he falls as in epilepsy; general convulsions follow, the eyes are turned upward or inward, the pupils are contracted for a few moments, then dilated, and remain so during the attack, the face is flushed or purple, head hot, jugulars distended, pulse strong and frequent, the jaws are closed, respiration hurried and imperfect, but not stertorous. I have not noticed any involuntary discharges, or spasm of the glottis.

This state of convulsions may last from one to five minutes, followed by perfect coma or a partial return of sensibility, which lasts from five minutes to an hour, when another attack comes on, followed again by more perfect and persistent coma, which continues in the worst stages until death closes the scene, or it may again be interrupted, at intervals, by convulsions. In the mildest cases patients recover without treatment, after one or two convulsions, as the hot stage subsides; but some squinting and nervous agitation may then be remarked for ten or fifteen hours, and a recurrence of the chill is almost certainly followed by severe convulsions.

The successful treatment of these cases, when commenced early, is sufficiently easy. Regarding the condition of the brain as one of pure congestion, whatever may be the cause, with a tendency to terminate speedily in effusion, the *rationale* of the following treatment is as plain as it is successful and easy, when commenced before such change has occurred in this delicate organ as is almost necessarily fatal. The following cases will serve to illustrate the treatment, better perhaps than a formal discussion.

R. J. A robust male child of healthy parents, æt. two years, had had ague in the spring of '47 without complications, from which he had perfectly recovered. On the 15th August following, about mid-day, he had a mild paroxysm of intermittent fever, marked by nothing unusual, except some slight nervous agitation during the hot stage. Having had no medicine on the 17th, at noon the usual signs of a chill came on, and lasted nearly half an hour, when it subsided, and in about five minutes after, severe general convulsions suddenly commenced. Being present at that moment, I had his head held over a vessel, and commenced at once pouring cold water in a full stream from a height of ten or twelve inches, on the neck and head: in less than a minute all spasmodic action had ceased, the face and neck which had seemed distended with blood, were now pale and cold, but the child was insensible. I continued the application less profusely, and in three or four minutes he gave evidence of improvement, by crying and struggling against it. The douche was now abandoned, but the head was kept wet with the water, during the continuance of the hot stage. No other remedy was used, the case assumed the usual appearances of a paroxysm of ague, was treated as such, and speedily recovered. This child has had intermittents since, and on one or two occasions there has been some spasm of the extremities during the hot stage, but a quart of water fresh from the well, poured on the head, always prevents further mischief.

Sarah B., a healthy, but rather slender child aged four and a half years, was seized in Sept. '46, with general convulsions during the formation of the hot stage of a fit of ague. I saw her in fifteen minutes after their commencement, and found her in an imperfectly comatose state, head hot, pupils slightly dilated and immovable, face looked full, but the expression was placid, the convulsion having subsided. As sensation was not entirely abolished, she could still swallow, I then gave her about six grains of Ipecac, and placed her feet and legs in hot water ; the convulsion again commenced. Cold water was now freely applied by the douche as in the first case, all spasm subsided, and by a continuance of it, the little patient became sensible in about five minutes. Shortly after she vomited freely, the skin became moist and in an hour all the head symptoms had subsided. She was immediately put on the use of Sulph. Quinine, and recovered without further bad symptoms.

September 6th, 1846 was called to see a male child aged nine months, of previous good health, parents healthy. Found on arriving that the child had had nearly four hours before a well marked chill, apparently of ague, which had excited no alarm as the disease was very prevalent at the time. On the subsidence of the chill, which lasted some 40 minutes, convulsions commenced and lasted about 2 minutes: this state was succeeded by stupor nearly perfect, of about half an hours duration, this was followed by another convulsion to which a comatose state again succeeded. In this way convulsions and coma alternated until the time of my arrival, a little more than three hours after the first fit. Perfect insensibility was then present, muscular system relaxed, pupils dilated, extremities and surface generally warm, pulse 120 rather feeble, no fullness of jugulars, face pale. Nothing had been done for the child except the application of spts. turpentine to the chest and immersing it in warm water. I applied for a few moments the cold water in a stream to the head, but finding it reduce the force of the heart's action, I desisted but kept the part wet with it. Mustard plasters were applied to the back of the neck, abdomen and legs, and a stimulating enema exhibited. But those efforts were of no avail; the disease had advanced too far, the pulse continued to fail, respiration became embarrassed, and the patient sank without further convulsion in about two hours, being about five and a half hours after the first fit. I regret that a post-mortem examination could not be had.

This is the only case of the disease which I have seen terminate fatally, but it is enough to prove the necessity for active treatment, to ward off the tendency to effusion which seems here evidently to have taken place as a result of the congestion.

These cases, selected from a number of others, will be sufficient in illustration. There is nothing new in the treatment, consisting as it does mainly in the use of usual revulsives and the powerful refrigerant, the cold water douche, which seems to me to have been too much neglected in the treatment of a cerebral disease, especially of children, when the attacks are often sudden, with a tendency to a speedy fatal termination. The remedy is a powerful one especially when assisted by a warm semi-capium, and I can truly say I have not seen it fail to relieve infantile convulsions, except such as arose from exhaustion, or when the cause was remote from the brain.

I may state in conclusion my impression that in these convulsions of children, the condition of the brain is allied to that of puerperal patients. In one case the excitable state of the brain being common to the period of childhood, in the other, an irritable and excitable condition of the nervous system generally accompanying pregnancy and parturition: hence convulsions occur in either case, strikingly similar in their general features, excited by causes which, in in a different state of the nervous centre would give rise to no such manifestations, which are relieved by the same class of remedies speedily, and without which treatment, in either case, death would often quickly ensue. It is true, bleeding is seldom needed, though often used in treating the infantile disease; for cold water applied as a douche, is equally speedy and powerful, and much more easily had recourse to, and children will recover from its effects almost immediately, who would not rally entirely from the loss of three or four ounces of blood in as many days.

